# Path Planning for Non-Circular, Non-Holonomic Robots in Highly Cluttered Environments

**DOI:** 10.3390/s17081876

**Published:** 2017-08-15

**Authors:** Ricardo Samaniego, Joaquin Lopez, Fernando Vazquez

**Affiliations:** 1 Imatia Innovation, 36310 Vigo, Spain; 2 Department of Systems Engineering and Automation, School of Industrial Engineering, University of Vigo, 36310 Vigo, Spain; joaquin@uvigo.es (J.L.); fvazquez@uvigo.es (F.V.)

**Keywords:** mobile robots, car-like robots, non-holonomic, path planning, motion planning, state lattice

## Abstract

This paper presents an algorithm for finding a solution to the problem of planning a feasible path for a slender autonomous mobile robot in a large and cluttered environment. The presented approach is based on performing a graph search on a kinodynamic-feasible lattice state space of high resolution; however, the technique is applicable to many search algorithms. With the purpose of allowing the algorithm to consider paths that take the robot through narrow passes and close to obstacles, high resolutions are used for the lattice space and the control set. This introduces new challenges because one of the most computationally expensive parts of path search based planning algorithms is calculating the cost of each one of the actions or steps that could potentially be part of the trajectory. The reason for this is that the evaluation of each one of these actions involves convolving the robot’s footprint with a portion of a local map to evaluate the possibility of a collision, an operation that grows exponentially as the resolution is increased. The novel approach presented here reduces the need for these convolutions by using a set of offline precomputed maps that are updated, by means of a partial convolution, as new information arrives from sensors or other sources. Not only does this improve run-time performance, but it also provides support for dynamic search in changing environments. A set of alternative fast convolution methods are also proposed, depending on whether the environment is cluttered with obstacles or not. Finally, we provide both theoretical and experimental results from different experiments and applications.

## 1. Introduction

The problem of planning a path for a geometrically and dynamically constrained mobile robot in a cluttered environment is one of the most time-consuming tasks in the field of mobile robotics. A common approach is to search for the path over a grid-based cost map, in which each cell has a value ranging from zero (free space) to a very high value representing non traversable areas (MAXCOST). Intermediate values represent either high cost movements (e.g., difficult terrain) or areas where there is a high probability of collision, making it risky to move at the robot’s normal speed and probably forcing it to slow down. Search is performed in a step-by-step manner, starting at the cell where the path begins, evaluating the cost of moving the robot to each adjacent cell, selecting the action with the lowest cost, moving the robot to that cell and from there repeating the process in a recursive or iterative manner until an optimal path to the target location is found. To evaluate each of the possible actions, even if they are finally discarded, the algorithm must perform a convolution between the robot’s footprint, with its anchor point (typically the geometric center) placed on the evaluated cell and the cost map. Typically, the maximum value of this convolution is taken into account to evaluate the cost of the potential action. This convolution is computationally expensive when a high resolution is used to model the robot’s footprint and the local map’s features. Furthermore, if the paths to be found are long and a high spacial resolution is also used for determining the steps, the number of convolutions to be performed, and thus the computations, grows exponentially.

A common approach to reduce the complexity of this problem is to approximate the robot’s footprint to its enclosing circle, precomputing a cost map in which obstacles are inflated by the radius of this circle, effectively reducing the robot to a single point and avoiding the need to perform convolutions when searching for the path. This approach works well for robotic vehicles that are approximately circular or small compared to the environment and obstacles in which they move, but not so for slender (elongated) vehicles that must move in environments in which they are large compared to the obstacles and the free space through which they must move. For these types of vehicles, approximating them by their enclosing circle makes them look much larger than they actually are, and turns out to be too conservative for most practical uses. As a result, narrow passages are often considered non traversable even when they would actually be feasible for certain orientations or configurations of the robot. For these non circular robots, planning using the actual footprint implies a real-time convolution for every single action on a three-dimensional space (*x*, *y* and orientation). For example, for a 5 m × 2 m robot on a grid of 0.1 m resolution, a single 5 m forward movement involves the evaluation of about 2000 cells.

In this paper, we present a new approach to the problem, in which a set of precomputed maps are used, convolving the footprint of the robot with the entire map for a set of possible orientations, discretizing the map for *x*, *y* and orientation. The method reduces any robot, regardless of its footprint, to a single point that must traverse a 3D precomputed map, thus avoiding the need of any real-time convolutions. For applications in which dynamic search is required, normally due to a changing environment, we propose carrying out a local convolution, whenever new information regarding the environment is received, to update the precomputed 3D map. To do that, we propose using fast convolution methods based on the Fast Fourier Transform (FFT) or the morphology dilation operation, depending on whether the environment is cluttered with obstacles or not.

The rest of the paper is organized as follows. First, a brief description of the prior work in this area is provided in the next section. Then, the key concepts and techniques of our solution are described. Section 4 includes a description of the different methods used to obtain the convolution of the robot with the local map, including an analysis of the complexity involved. The description of the graph search algorithms that we have used is included in Section 5. Finally, we present theoretical results in simulated environments as well as experimental results obtained from an implementation for a large “tugmaster” articulated truck navigating inside a narrow warehouse.

## 2. Related Work

Autonomous path planning and navigation of non-holonomic vehicles have been an active field of research during the past decades and several surveys about this topic have also been published [1]. Robotic developments are increasingly moving from small, circular research robots to vehicles that are large and fast such as cars or trucks. When it comes to motion planning, small circular robots have the advantage that they can stop almost instantly and can turn in place if they are equipped with differential drives, making the path planning problem for them very close to that of a holonomic vehicle. Large vehicles pose many more challenges for path planning algorithms due to their dynamic and spatial characteristics, including a limited turning radius, as well as the fact that they tend to be slender or elongated, rather than circular or almost circular.

Early approaches focused on local planning, where only short term paths are calculated to navigate towards a given goal while avoiding obstacles, whether previously known or detected in real time [2,3]. This local approach is prone to get stuck on convex obstacles, such as cul-de-sacs or other forms of local minima, and it is also incapable of reasoning complex maneuvers for navigating in complicated environments such as sharp turns on narrow corridors or U-turns. Much of the recent work is focused on global path planning, taking the robot from the current position to a given goal, reasoning a complete path that is compatible with the restrictions posed by the vehicle and the environment [4]. This global path is usually computed through the concatenation of minor discrete actions taken from a precalculated control set, forming a state lattice [5]. These actions connect different states of a discretized space, allowing the path planning problem to be formulated as a graph search. This global approach is very challenging in terms of memory consumption and processing time, especially when high resolutions are needed.

This approximation discretizes the space in a grid on which each cell is a state of the lattice which describes the corresponding location of the map, so it is possible to define different costs for each cell. If the vehicle dimensions are not negligible with respect to the size of the cell, this method will not address the problem of collision with a nearby object. A common practice for robots of cylindrical or near-cylindrical shape is to grow the obstacle by the radius of the robot itself [6], but this approximation is not appropriate for slender robots.

Several approaches have been made to address the problem of finding a path for an arbitrarily shaped robot in the presence of obstacles. The most common approach is the online calculation of collisions at each step of the graph search algorithm by convolving the footprint of the robot with the map, having rotated the footprint to the corresponding angle of the maneuver in progress [4]. Furthermore, as each individual motion primitive of the control set can cross several cells of the state space, a set of finely spaced intermediate poses is calculated for each of these primitives. All of these poses must be checked to ensure that a given motion is valid between the two states. Although several optimizations and precalculations can be made [7], the computation time of these convolutions grows rapidly with map resolution and the density of obstacles.

In environments where the density of obstacles is high, one approach is to reduce the resolution in order to limit the total calculation time, but this implies increasing the padding distances to ensure that the robot will always be at a safe distance from the obstacles found throughout the path. This makes it impossible for the robot to be positioned near walls and, in environments with narrow corridors, can eventually prevent the robot from finding a suitable path to the goal.

Likhachev and Ferguson [7] propose the use of two preconvolved obstacle maps in order to reduce the calculation time: one with the obstacles grown by the radius of the circle circumscribed to the robot and another one with the inscribed circle. This way, a motion primitive that is successfully checked against the first map is guaranteed not to collide in any possible heading, while a motion primitive overlapping an obstacle on the second map will not be feasible for any orientation. Only the paths traversing an obstacle cell on the first map but not on the second one will require a convolution between the real obstacles’ map and the vehicle’s footprint, in order to ensure a collision-free trajectory. However, in an environment with a high density of obstacles, a detailed convolution can be expected to be necessary for the vast majority of steps, making the performance improvement provided by using these two additional preconvolved maps insignificant.

Another optimization is made in the same paper, with the use of two separate action spaces, of two different resolutions. The paper proposes using the high resolution action space in the vicinity of the robot and the goal, while using the low resolution action space elsewhere. As the computational cost of the graph search increases with the size of the action space, as well with the size of the occupancy map, the idea is that the use of this low resolution action space for the majority of calculations will reduce the total calculation time. However, the resolution of the action state, is not always critical only around these two points, but also in cluttered zones where the robot has to maneuver closely in order to pass between obstacles. Considering the problem that we are addressing in this paper, where we are not generally assuming the presence of large clearance zones between obstacles, this previous approach would result in a permanent utilization of the high resolution lattice, without accessing the benefits of using the one of low resolution.

An efficient grid-based spatial representation to compute dynamic layered c-space maps is proposed in [8]. However, the solution includes a path planning method that is restricted to robots without a non-holonomic constraint because they do not use a state lattice.

One of the most interesting approaches that has been made, for the purposes of the problem addressed in this paper, is the utilization of two different occupancy maps: the original obstacles map and a second one inflated by the radius of a set of circles inscribed in the robot’s footprint [9]. The points of the footprint are then divided into two sets: points that should be evaluated against the cost map that has the inflated obstacles and points that should be evaluated against the original cost map. Although the online convolution of the footprint is not completely avoided, the number of points to be convolved in each step is greatly reduced. In a real case scenario, the authors show that more than 60% of the calculations can be avoided. However, for large robots in high resolution scenarios, convolving 40% of the footprint cells still implies an unacceptably high computation time.

A totally different approach to the problem is made by sampling-based planning methods [10,11]. These methods discretize the continuous space by taking samples on the fly, without using a previouslly existing graph representation of the search space. While these methods are usually much faster than the search-based ones, they do not guarantee the optimality of the results, providing inefficient paths. Examples of this methods are RRT [12] or PRM [13]. More recent works have extended these techniques in order to achieve probabilistic completeness, or even asymtotic optimality [14,15,16]. However, none of these sampling-based methods can guarantee the optimality of the generated path.

In this paper, we present a novel, search-based, path planning method that is especially advantageous in the case of elongated or slender vehicles that must operate in heavily cluttered environments. This method assumes that the map, obstacles and shape of the robot are previously known and precomputes a 3D obstacles map, where the first two dimensions represent a discretized horizontal space and the third dimension represents the robot’s pose or orientation. Once the 3D map is constructed, the search for an optimal path between any two points can be performed as if the robot occupied a single point in this pseudo 3D space and without having to perform costly convolutions at run-time. To support moderately changing environments, we propose partially updating the 3D map by means of applying a Fast Fourier Transfer (FFT) or simple morphology dilation operations, depending on the type of occupancy map that is available.

## 3. Algorithm Basics

The traditional approach that has been used for planning paths to be traversed by ground vehicles, in environments that have been previously mapped, is to use a 2D cost map that represents the distribution of the obstacles. Although the occupancy maps used for motion planning are two-dimensional, searching for a minimal cost solution, when the vehicle is non cylindrical, requires considering three dimensions (*x*, *y* and orientation). This three-dimensional look up is converted into a discrete set of two dimensional checks, which must be done in real time and require a highly time-consuming convolution operation.

Let us first introduce the notation that is going to be used in the rest of the paper. The workspace W is the map of the robot’s working area. In our case, $W\subset R^{2}$ is discretized into an array *W* of n×m cells. In a binary map, each cell (W(i,j)) can only have two possible values, representing an obstacle (W(i,j)=1) or free space (W(i,j)=0), while, in a cost map, each cell value represents the cost of traversing the cell (W(i,j)⊂R).

The robot is a rigid body of any shape and the **robot footprint** $A\subset R^{2}$ is the set of all points of W that lie in A.

The **robot configuration** *q* is a minimal set of parameters that specify the position of the robot. For our application, we employ a three-dimensional (x,y,θ) representation where (x,y) represents the position and θ represents the vehicle’s orientation. The **configuration space** of a robot (**C-space**) is the set of all the possible configurations.

For a robot configuration q(x,y,θ), we will represent the robot’s footprint A as $A_{\left(x,y,\theta \right)}$. In addition, we define the footprint matrix $A_{\left(x,y,\theta \right)}$ as a set of cells (i,j) with the same size as the cells in the workspace *W*, according to the following equation:

(1) $$A_{\left(x,y,\theta \right)}\left(i,j\right)=\left\{\begin{array}{cc} 1, & \;\mathrm{if}\;A\;\mathrm{overlaps}\;\mathrm{the}\;\mathrm{cell}(i,j),\\ 0, & \;\mathrm{if}\;A\;\mathrm{does}\;\mathrm{not}\;\mathrm{overlap}\;\mathrm{cell}(i,j), \end{array}\right.$$

where the dimensions of A (a×b) will depend on the size of the robot and the resolution of the workspace *W*.

**Figure 1:**
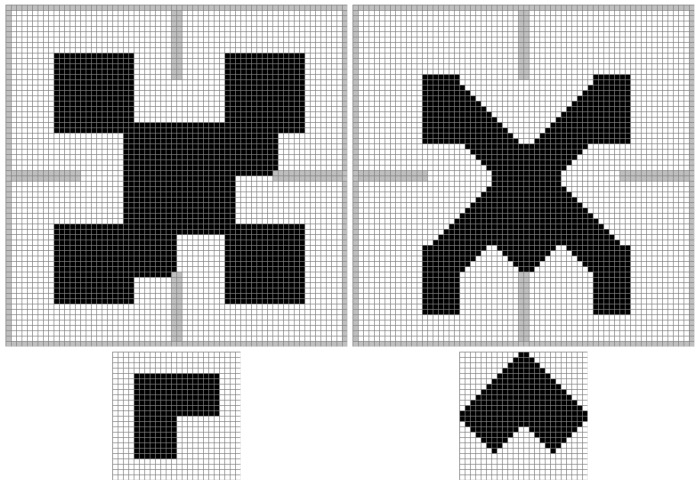
Detail of two layers of convolution.

The bottom part of Figure 1 shows the representation of the footprint matrix ($A_{\left(0,0,\theta \right)}$) of a L-shaped robot for two different orientations (θ).

**Figure 2:**
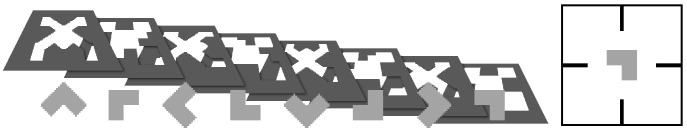
Preconvolved 3D occupancy map.

We present an algorithm that completely avoids the need for real-time convolution calculations by effectively performing three-dimensional checks, using a precomputed set of inflated cost maps. In regular occupancy maps, the discretization of the space is made in two dimensions (x,y), but here we propose to also discretize the orientation taken by the vehicle, inflating the map with the actual footprint of the robot for each discrete angle value, thus generating a set of preconvolved maps. Thus, any cost computation and collision checking can be done in real time by just taking the appropriate value from this set of spatially inflated maps. The resulting data can be seen as a single 3D map, in which the *z*-coordinate represents the orientation of the robot. More specifically, this map will be toroid-shaped, given the fact that the top and bottom layers of the 3D map are actually connected. An example of this 3D map is shown in Figure 2, where a 128 × 128 cells binary map is converted to an eight layer 3D map, corresponding to an angular resolution of 45 degrees.

In Figure 2, the original binary map is on the right, with obstacles represented in black and free space in white. The robot’s footprint is shown over the map in light gray. On the left, the layers of the inflated map are shown, along with the rotated footprint used for the convolution of each layer. In Figure 1, two layers of this convolution are shown in detail, along with the corresponding rotated footprint.

## 4. Obtaining the Cost for Each Action

When generating the inflated maps, we take a slightly different approach for binary maps than for non-binary (grey) cost maps. Non-binary cost maps represent cells that could be potentially traversed, albeit with a certain probability of collission or difficulty, so our approach is to convolve the robot’s binary footprint, for each discrete orientation value, with each one of the map’s cells. In contrast, binary cost maps represent cells that are either occupied or free, so we propose using a morphology dilation operation for these.

### 4.1. Obtaining the Cost through Convolution

Therefore, for the the non-binary situation, we perform a regular matrix convolution, in which overlapping elements are multiplied and the results are summed up. This means that the cost of being at a certain position (x,y,θ) can be obtained as the sum of cost of the cells overlapping the robot’s footprint A:

(2) $$C\left(x,y,\theta \right)=\sum_{\begin{array}{c} x-\frac{a}{2}<i<x+\frac{a}{2}\\ y-\frac{b}{2}<j<y+\frac{b}{2} \end{array}}W\left(i,j\right)A_{\left(x,y,\theta \right)}\left(i,j\right).$$

For the moment, any problems that might arise concerning the range of the indexes in the borders have been ignored. However, when obtaining the costs as in Equation (2), indexes *i* and *j* that are beyond the borders (i<0, i>m, j<0 and j>n) should not be considered. From a theoretical point of view, we can assume that *W* and *F* are padded with zeros in all directions.

For a fixed orientation θ, the footprint matrix for a position $A_{\left(x,y,\theta \right)}$ can be obtained as a translation of the footprint matrix for another position $A_{\left(0,0,\theta \right)}$ and Equation (2) can be reformulated as:

(3) $$C\left(x,y,\theta \right)=\sum_{i,j}W\left(i,j\right)A_{\left(0,0,\theta \right)}\left(i+x,j+y\right).$$

The convolution of two arrays (E*B) can be obtained with the equation:

(4) $$\left(E*B\right)\left(x,y\right)=\sum_{i,j}E\left(i,j\right)B\left(x-i,y-j\right).$$

If we compare the terms in Equation (3) with this equation, we can realize that, for a fixed θ, we can obtain the cost as:

(5) $$C\left(x,y,\theta \right)=W*A^{\prime },$$

where, as proposed in [17], $A^{\prime }$ is defined as (There is a shift in the position for which the cost is obtained of $\left(\frac{a}{2},\frac{b}{2}\right)$ because element F(0,0) is not the center of the robot):

$$A_{\theta }^{\prime }\left(i,j\right)=A_{\left(0,0,\theta \right)}\left(-i,-j\right).$$

After the convolution, a normalization step is performed, in order to obtain costs that are consistent all over the map, regardless of the robot’s footprint size. Furthermore, when the robot’s footprint overlaps a non traversable cell, the cell cost is set to MAXCOST, instead of the actual convolution result. This prevents a small robot with a pose overlapping an obstacle to have a lower cost than a larger robot with a pose that takes it over rough but traversable terrain.

### 4.2. Obtaining the Cost through Using a FFT

The convolution theorem states that under suitable conditions the Fourier transform of a convolution is the point-wise product of the corresponding Fourier transforms. Therefore, in our case, we can obtain the cost as:

(6) $$C\left(x,y,\theta \right)=W*A^{\prime }=F^{-1}\left\{F\left\{W\right\}⊙F\left\{A^{\prime }\right\}\right\},$$

where F{W} denotes the Fourier transform of *W* and E⊙B denotes the point-wise product of both matrices. An obvious advantage of this solution is that the use of the FFT allows us to reduce computation time. However, in order to apply the FFT to Equation (5), both matrices $A^{\prime }$ and *W* must have the same dimension because both “functions” are assumed to be periodic with the same period as if the matrix were to repeat periodically. A possible solution is to pad the $A^{\prime }$ matrix with zeros. We use the C library FFTW [18] to implement the FFT.

### 4.3. Obtaining the Cost through a Morphology Dilation Operation

When dealing with binary occupancy maps, where just traversable/occupied values are recorded, a morphology dilation operation [19] can be used to obtain the binary convolution map using the robot’s footprint as the kernel.

The morphology dilation operation uses the robot’s footprint as the kernel of the operation, but mirroring it both horizontally and vertically. This technique produces the same result as a regular convolution while taking much less computation time, especially when dealing with clear maps that have few obstacles, mainly because the computational effort is only required for obstacle cells and not for those that represent free space.

The workspace W is represented as the set of cells occupied by obstacles:

W={(i,j)/W(i,j)=1}.

To simplify the notation, we’ll use a single variable for a coordinate pair, w=(i,j). The robot’s footprint A in this case is going to be centered at the origin (0,0) and mirrored horizontally and vertically. Then, it is also represented by a set of points:

A={(i,j)/A(i,j)=1}.

The morphology dilation operation is going to obtain a new set:

C=W⊕A={(q+a)∀q∈W,a∈A}.

The result will represent the occupied cells after the convolution of the map and the robot.

### 4.4. Complexity Analysis

The convolution of the robot’s footprint and the map for each orientation is carried out offline, as it does not need to be a real-time operation. However, as will be discussed later, for dynamic graph planners, a local convolution must be calculated every time the cost of relevant map cells is updated. For example, when a new obstacle is detected, the cost of the cells where the obstacle is located should be increased and the planner should plan a new path to avoid a collision with the obstacle. Therefore, it is important to analyze the complexity of the different methods to obtain the cost of the actions in order to determine which of these methods is faster in each particular case. The first point that should be noticed is that the morphology dilation operation, as described here, can only be applied to binary maps.

The morphology dilation operation method is found to be the fastest method in environments with very few obstacles. However, as the map gets cluttered with more obstacles, the complexity of the dilation operation method gets closer to that of the convolution method.

The complexity of Fast Fourier Transform (FFT) is on the order of O(NlogN) operations [20], where *N* is the size of the data. The two-dimensional FFT requires taking the FFTs in each row and then in each column. Therefore, the complexity of a matrix M×N is O(NMlogM+MNlogN)=O(MN(LogM+logN)). Considering a square environment, the complexity is $O(N^{2}logN)$ operations. When directly applying the convolution, the complexity is $O(N^{4})$. It looks like the FFT saves quite some time. However, to be able to compare the complexity of both methods, we need to take into account the following issues:

- For the FFT method, it is necessary to calculate the workspace’s FFT (F{W}), the robot footprint’s FFT (F{A}), the point-wise product of both matrices and the inverse FFT of the result. The C library FFTW [18] has been used to implement the FFT transforms.
- For the FFT method, the robot’s footprint matrix *A* has been padded with zeros to match the dimension of the workspace (*W*) increasing the complexity of the FFT. That means that as the difference in size between *W* and *A* increases, the the FFT method is going to be less advantageous.
- When obtaining the cost map for each robot orientation (θ), it is only necessary to calculate the workspace’s FFT once (F{W}).

**Figure 3:**
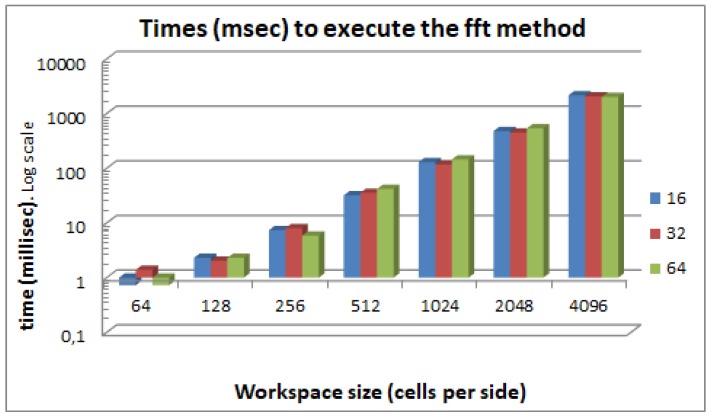
FFT method execution times for different robot and workspace sizes. The robot and workspace are square and the size is the number of cells on each side.

**Figure 4:**
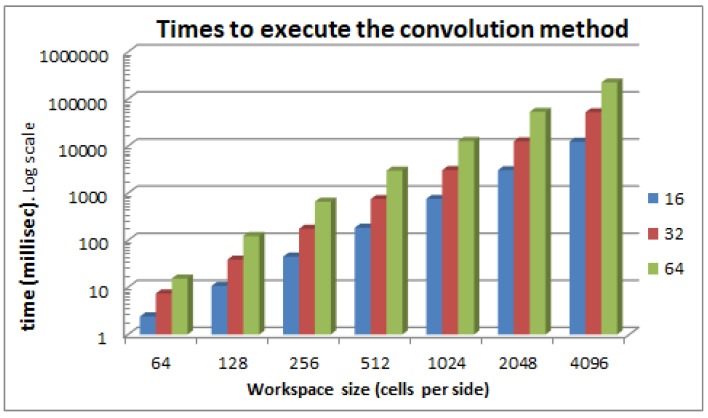
Convolution method execution times for different robot and workspace sizes. The robot and workspace are square and the size is the number of cells on each side.

**Figure 5:**
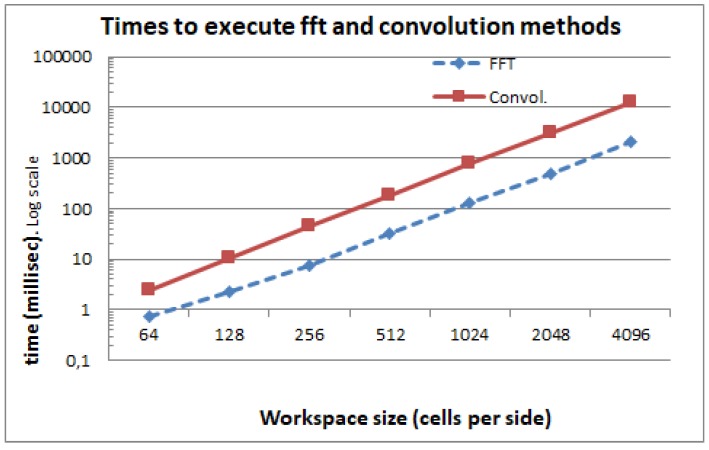
Comparison of the convolution and FFT execution times for a robot of 16 × 16 cells and varying workspace sizes.

Figure 3 shows the execution times of the FFT method for different sizes of *W* and *A*, while Figure 4 shows the execution times of the direct convolution method for the same configurations. As can be appreciated by comparing the two figures, the FFT method reduces the calculation time for all the cases. For a particular robot size *A* and varying workspace sizes *W*, Figure 5 shows that the FFT is always faster and that the difference just gets bigger as the size of the workspace increases. This is because the complexity of the FFT is O(NlogN) while the convolution is $O(N^{4})$, as we have seen before. Here, we do not present the execution times for the morphology dilation operation method because it mainly depends on the size of the robot’s footprint and the size of the obstacles. For very clear environments, it will be even faster that the FFT, while, for very cluttered environments, it will get closer to the pure convolution method.

## 5. Graph Search

Once the 3D map has been generated offline, the problem is reduced to a graph search on a three-dimensional space. Any graph search algorithm can be used in order to find a dimensionally feasible path, such as A* [21], ARA* [22] or ADA* [23].

We conducted our tests using the ARA* (Anytime Repairing A*) algorithm. This is an anytime heuristic search algorithm based on A*, which introduces a heuristic inflation technique (by a factor ϵ>1). Anytime planning algorithms find an initial, usually suboptimal, solution, which is then progressively improved until the optimal path is found or the allocated time expires. The advantage of ARA* is that it iteratively performs calculations with progressively lower ϵ, while reusing the previous search results in order to reduce the calculation time. In addition, this algorithm provides a bound on the sub-optimality of the solution, which is the ϵ factor itself.

When using replanning graph search algorithms, such as Anytime Dynamic A* (ADA*), a real-time convolution will be required for any newly found obstacles. This will also happen when using the technique based on the two inflated maps (inscribed and circunscribed). To update this 3D map, we will have to recalculate the cost of the cells corresponding to any orientation and located within a $\sqrt{a^{2}+b^{2}}$ distance of the ones that have changed, *a* and *b* being the length and the width of the convex hull of the robot’s footprint, respectively. For binary maps, this operation can be performed very fast by using the morphology dilation technique. For regular cost maps, the FFT approach will allow the update to be performed in a reasonable amount of time (usually less than 100 ms). We have already provided a study on the execution times for different robot and map sizes in Section 4.4.

In order to guarantee the kinodynamic feasibility of the computed path, a precomputed set of motions is used, along with a lattice-based planner [5]. By means of this lattice, the motion planning problem is resolved as a graph search and the precomputed motions guarantee that the path is traversable. In this case, as the space is discretized on a 3D map, the lattice is also calculated in three dimensions, with the third dimension representing the orientation of the robot. Thus, a turn in place operation is represented as a vertical connection between equal *x* and *y* coordinate cells, with the start of the connection at the original vehicle orientation and the end at the final one. To achieve kinodynamic feasibility, these precomputed motions need to accomplish the following:

- The curvature on every point of the motion has to conform with the robot’s minimum turning radius.
- There must not be discontinuities regarding the position (jumps), orientation (sharp turns) or curvature (steering wheel jumps).

**Figure 6:**
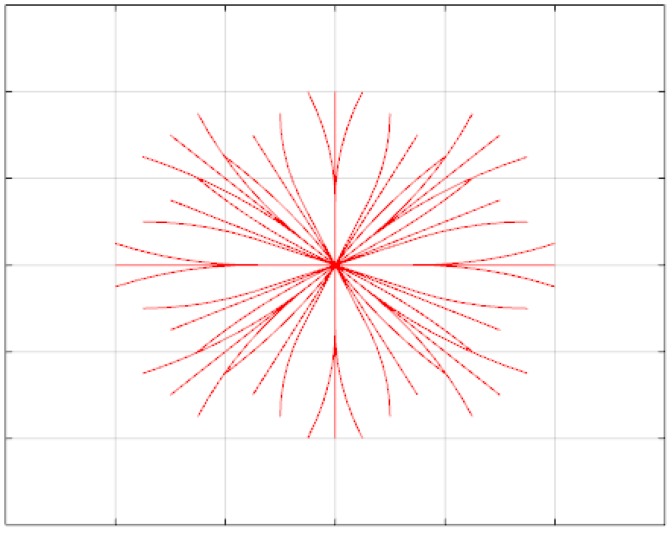
Motion set of Euler spiral primitives.

In order to accomplish these restrictions, Euler spirals are used for these motion primitives. These curves of continuous curvature are calculated offline [24] using the techniques described in [5] for achieving a reduced set. These motion primitives are then discretized, both in position and orientation, obtaining the list of 3D cells traversed by each motion, thus speeding up the online graph search. An example of these motion primitives set can be seen in Figure 6.

It should be noted that these precomputed discretizations will produce slightly different values than the ones calculated in real time. In the real-time approximation, a record of discretized occupied cells is precomputed for each exact pose value. In the proposed approach, the pose value is first discretized, and then checked against a precomputed map. Especially for large and slender robot footprints, this previous orientation discretization leads to a greater position error on the perimeter cells of the robot. This inconvenience for slender robots can be solved by incrementing the number of discretized orientations. In the proposed approach, experimental tests were conducted on a vehicle with a length of 12 m, for which 32 different discretized angles were needed in order to allow the robot to make turns at a distance of less than 0.2 m from large walls. We have found that similar results can be obtained with just 16 discretized angle values using the real-time approach.

In order to accomplish the aforementioned kinodynamic restrictions while performing the graph search, the motion primitive starting at each traversed cell must have an initial curvature that is similar to the final curvature of the previous motion primitive ending at that same cell. Note that it is not necessary to check that the orientations are equal at calculation time, since this is already represented by the actual 3D cell itself.

Although it could also be possible to require a continuity in speed when calculating the path, for low speed applications the effects of inertia may be neglected at the global planning level, delegating the task of managing these small errors to the robot’s local planner during navigation.

## 6. Comparative Analysis

**Figure 7:**
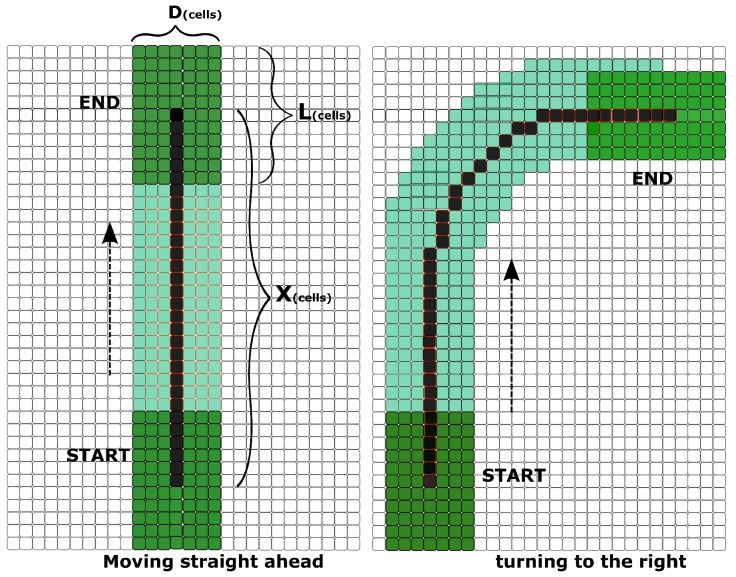
Cells covered by the robot while moving. On the figure on the left, the robot moves straight ahead. On the figure on the right, the robot turns to the right.

In Section 4.4, we have analyzed the complexity of the different methods proposed for the convolution of the geometric footprint of the robot with the map. There is a great interest in reducing this complexity because the most time-consuming part of global planning is computing the cost of each action *a*. Some approaches such as [7] implement some efficient convolution steps offline in order to reduce the convolution time needed to obtain the cost of action *a*. In that approach, for each action *a*, they precompute the cells covered by the vehicle when executing action *a*. Every time they need to evaluate action *a*, all of those cells are iterated to obtain the action’s cost. Figure 7 left shows the number of cells covered by the robot while executing the short action of moving 1 m straight ahead. In our approach, we precompute the convolution and store the value in the robot’s center cell. In the example of Figure 7, we show that in our approach we only have to iterate on the black cells, compared to former approaches where all the green and black cells had to be iterated. When the robot is moving straight ahead *X* cells (left part of Figure 7), the number of cells covered is X×D+FP (black, dark green and clear green), where FP=D×L are the robot’s footprint cells. On the other side, the cells needed to iterate if we store the convolution are *X* (black cells). When the precomputed convolution cost map is used, the number of cells involved in the iteration required for executing action a is reduced by a factor of D=robotwidth/cellwidth, compared to the approach that precomputes the cells covered by the vehicle. When the robot is moving and rotating at the same time, it tends to cover more cells (right part of Figure 7), because it is longer than wider and when it turns, the rotation center is in the middle of the rear wheels.

For a typical vehicle size of 5.5 m × 2.5 m and a resolution of 0.25 m, a short move straight ahead of 2 m requires the iteration and collision checking of roughly 310 cells ((5.5/0.25)×(2.5/0.25)+(2/0.25)×(2.5/0.25)). However, if the orientation is discretized and the convolutions are precomputed offline as we propose here, it will only be necessary to iterate over about nine cells. In the next section, we will discuss the results of both algorithms for two different scenarios.

## 7. Experimental Results

First, some simulated experimental results will be shown to compare the results and computation time of this approach with respect to other approaches found in the literature. Then, we will introduce some applications where the proposed algorithms have been implemented and tested.

### 7.1. Simulation Results

We used the Search Based Planning Library (SBPL) (http://www.sbpl.net/, version 1.3.0, Carnegie Mellon University - Robotics Institute, Pittsburg, PA, USA) for performing comparative tests, as this library implements the techniques described in [7]. In SBPL, the discrete representations of a planning problem are implemented by “Environments”. We compared the results of the presented algorithm against one called “EnvironmentXYTHETALAT”, which implements the technique described in [7]. All of the tests were conducted with the same set of motion primitives and the same computer, equipped with an Intel^®^ Core^TM^ i7-4770 CPU processor, running at 3.40 GHz.

In this section, we use the term “Reference” when referring to the original method and “Proposed” when referring to the proposed one, where the orientation is encoded by the third dimension.

Two different tests were performed: first, in a small clear occupancy map, called “cubicle” and, second, in the large cluttered occupancy map of the Willow Garage Company (Palo Alto, CA, USA) facilities, called “willow”. Both maps are included with SBPL and 3D occupancy maps are generated for the tests, following the technique described in this paper. Both tests were performed using the implementation of the ARA* graph search algorithm included with the library, using eleven different values of epsilon (3.0, 2.8, 2.6, 2.4, 2.2, 2.0, 1.8, 1.6, 1.4, 1.2, 1.0).

Moreover, while the cubicle test is conducted for a robot with a small rectangular footprint of 0.3 × 1.0 m, the willow test is conducted for a robot with a large rectangular footprint of 0.5 × 2.0 m. This leads to two very different environments: an easy one, where the robot can occupy most of the positions on practically any possible orientation, and most of the paths pass by cells outside the circumscribed zone; and a hard one, where the robot can traverse most zones only for a reduced set of orientations, and where most of the collision checks need to be performed during planning time, since the robot will be inside the circumscribed radius zone.

**Figure 8:**
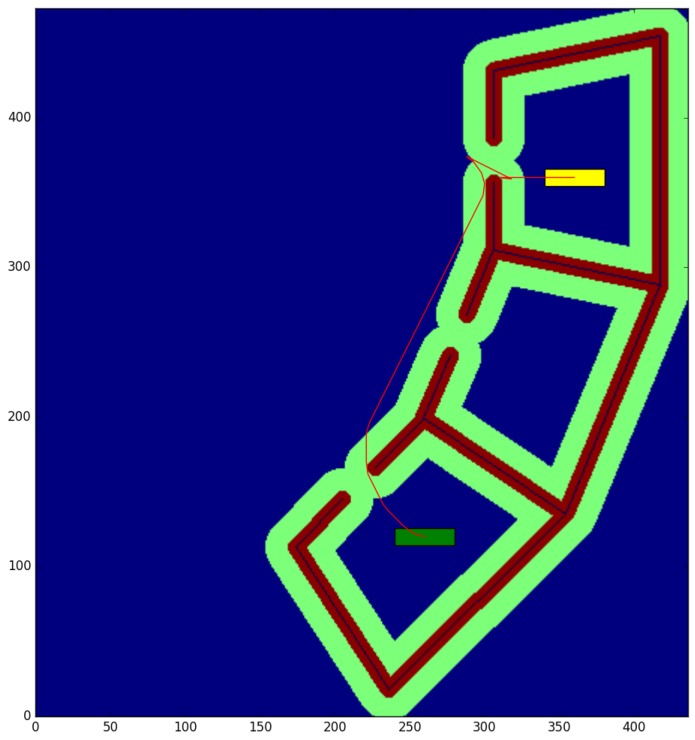
Reference method—Cubicle test.

**Figure 9:**
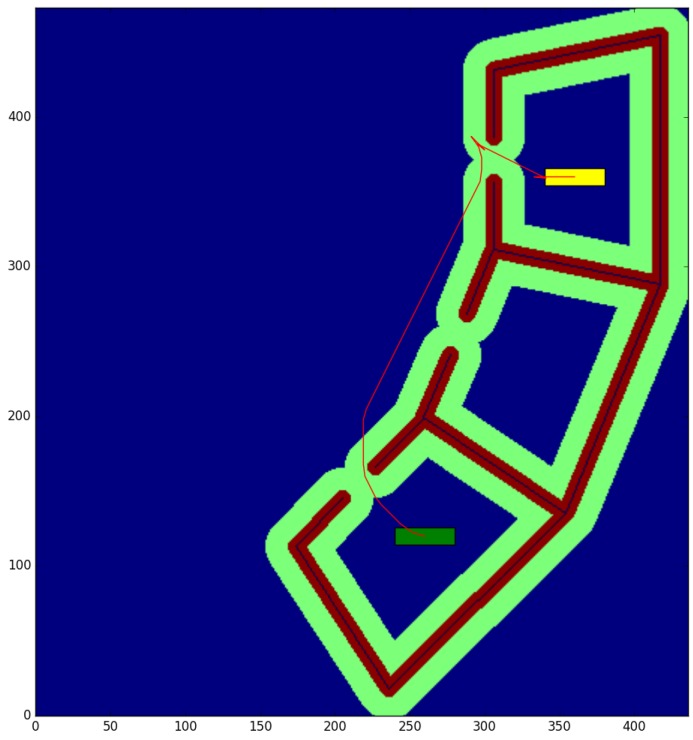
Proposed method—Cubicle test.

The cubicle test (Table 1) is conducted in a small environment of 10.9 × 11.825 m, with a resolution of 0.025 m per cell. We obtain the paths between the start and end points shown in Figure 8 and Figure 9, for a robot with a small rectangular footprint of 0.3 × 1.0 m. Both on the starting and ending positions, the robot is facing to the right. Since this map is very clear, the proposed method presents little advantage with respect to the one used as a reference (a time reduction of just 12.99%), as most of the path travels through clear zones, where obstacles are farther than the circumscribed radius. As it can be seen, the reduction in the average computation time per node is just 8.3%. A small path cost increase of 0.46% is due to the discretization errors that were previously described. These errors are also the cause for the 5.10% reduction in the number of visited nodes.

**Figure 10:**
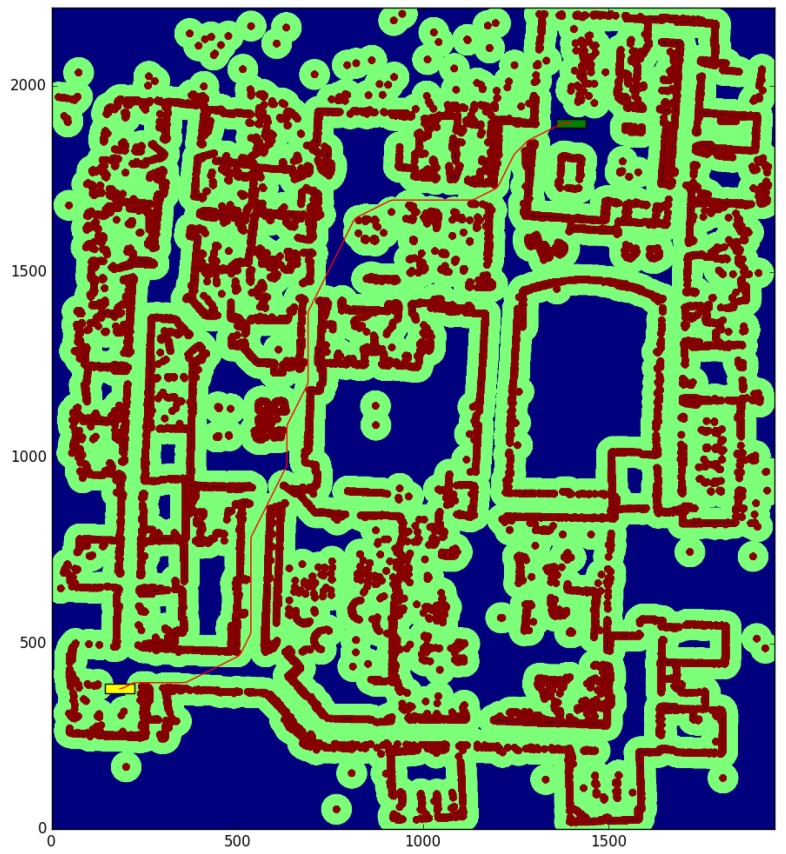
Reference method—Willow test.

**Figure 11:**
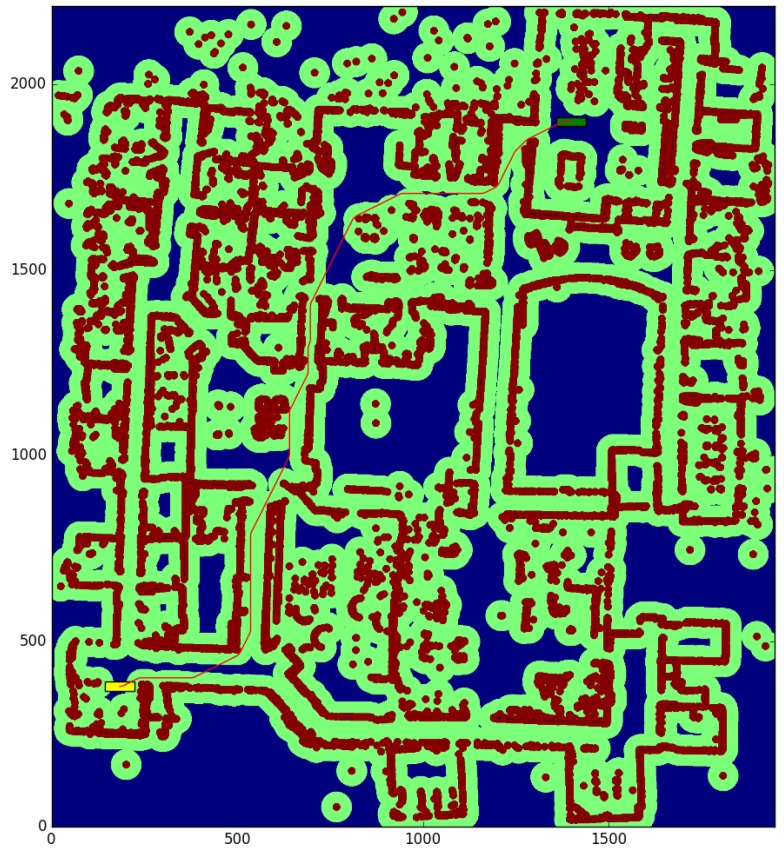
Proposed method—Willow test.

The willow test (Table 2) is conducted in a large environment of 48.675 × 55.275 m, with a resolution of 0.025 m per cell. We obtain the paths between the start and end points shown in Figure 10 and Figure 11, for a robot with a large rectangular footprint of 0.5 × 2.0 m. Both at the starting and ending positions, the robot is facing to the right. The environment is heavily cluttered compared with the robot’s size, so the proposed method presents great advantages with respect to the one used as a reference (a time reduction of 56.60%), as most of the paths must pass through cells where obstacles are inside the circumscribed radius. Now, the reduction in the average computation time per node is 28.0% Again, the path cost increase of 4.02% is due to the discretization errors, and these errors are also the cause of the 39.71% reduction in the number of visited nodes.

The cost for each path is calculated as the sum of the costs of all the motions conforming that path. The cost of each single motion is estimated as the time required to traverse it, considering for the conducted tests that the robot is capable of traveling at one meter per second, and these costs are weighted according to the following criteria:

- If the motion is completely straight, the cost is not altered.
- If the motion performs a curve, the cost is doubled.
- If the motion is traveling backwards, the cost is multiplied by five.

This approximation minimizes both the distance travelled backwards and the number of turns performed. This generates smoother paths that are easier to follow for a vehicle with slow and complex dynamics. This also reduces the effort and wear applied to the vehicle actuators (steering wheel, brake and accelerator).

The color code on the figures shown is the following:

- Blue: unknown cells.
- Black: untraversable (obstacle) cells.
- Green: cells invading the circunscribed radius of the robot (possibly in collision).
- Dark red: cells invading the inscribed radius of the robot (definitely in collision).
- Yellow rectangle: starting pose of the robot
- Green rectangle: ending pose of the robot.
- Red: calculated path.

### 7.2. Applications

**Figure 12:**
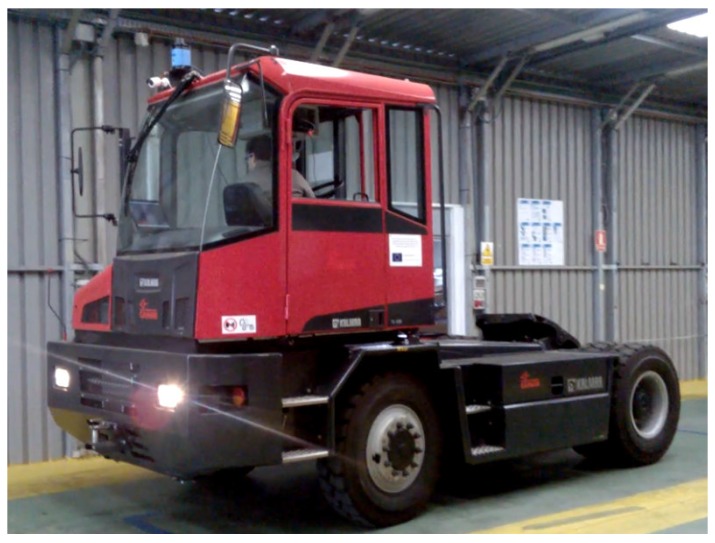
Robotized Tugmaster truck. Note the laser scanner for localization purposes on the top part of the cabin.

**Figure 13:**
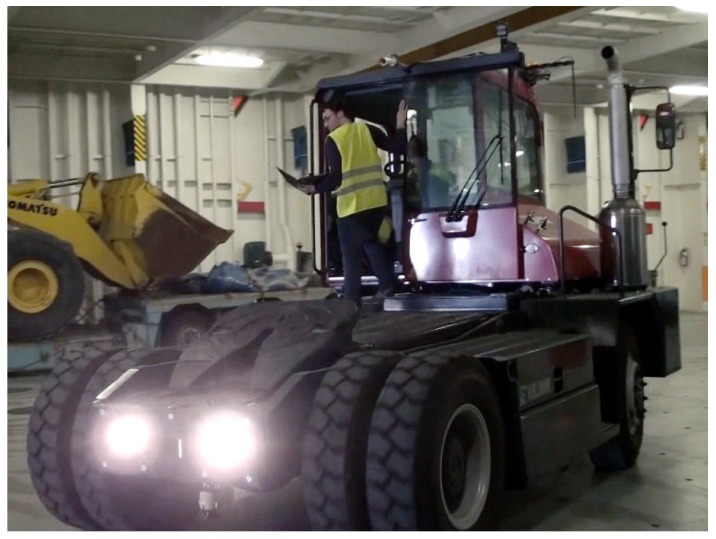
Robotized Tugmaster truck. Note the laser scanner for obstacles detection under the truck.

**Figure 14:**
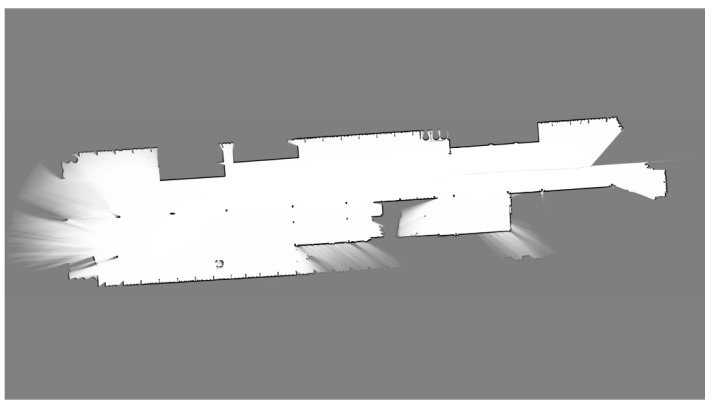
Ro-Ro ship hold map.

Real world experimental results were obtained with a robotized Kalmar Tugmaster truck (Figure 12 and Figure 13), in the framework of the Autoport project [25]. Here, the robotized vehicle was autonomouslly driven on a Ro-Ro ship hold, whose map is shown in Figure 14, as well as on a 9.5 m × 39.1 m narrow environment at a warehouse, which was also cluttered with several hydraulic jacks and other furniture. The truck had a width of 2.5 m and a length of 12.3 m.

Occupancy grid maps were constructed with a resolution of 2.5 cm using an implementation of Hähnel et al.’s map-builder algorithm [26]. This implementation builds a metric map from data recorded by a Sick NAV-350 laser-ranger. For that, it takes into account the distance and also the reflectivity provided by the laser readings. The map is stored as an occupancy grid and used by the path planning algorithm proposed here. Angular positions were discretized to 32 different values, thus obtaining an angular resolution of 11.25 degrees. Calculation times in this environment were as low as 0.6 s with ϵ=1.0 for the narrow warehouse.

The localization system integrates the laser-range and odometry readings using a variation of the Monte Carlo Localization [27] algorithm. Even though the Sick NAV-350 can determine the vehicle position with a high level of precision using three visible reflectors, we are using it in mixed-mode navigation mode that provides both spatial contour data and reflector data. We have chosen this mode because we cannot add new elements such as reflectors to the vessel.

**Figure 15:**
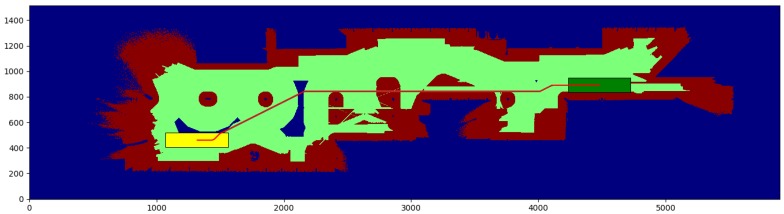
Reference method—Autoport test.

**Figure 16:**
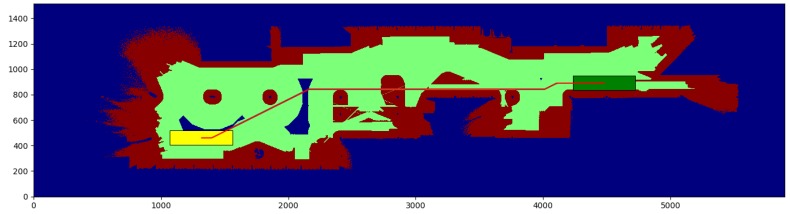
Proposed method—Autoport test.

We compared the proposed method with the one presented in [7] and implemented in SBPL, in the aforementioned ship hold. We used the same test conditions as in the previously exposed simulations but limited the path planning time to 180 s. The results are shown in Table 3. The reference method was not able to finish the search within the allocated time, thus obtaining a suboptimal path with an epsilon value of 1.2. The proposed method obtained the optimal solution (ϵ=1.0) in just 26.28 s. Both paths are shown in Figure 15 and Figure 16.

**Figure 17:**
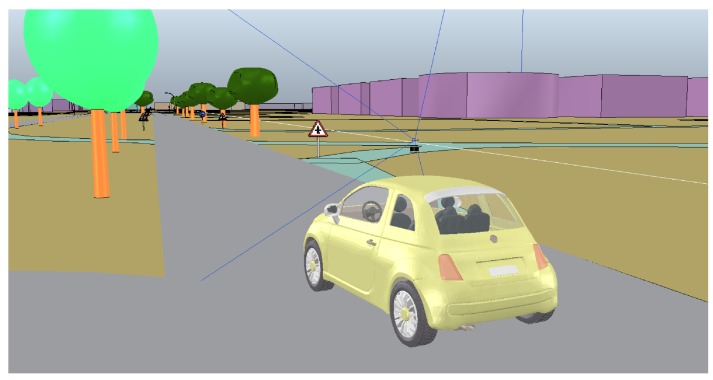
ElderlyCar project simulator (V-rep) in the testing environment of “Universidad de Alcalá”.

The approach presented in this paper is now also being applied to navigate an autonomous vehicle specially oriented for elders (SmartElderlyCar project). For this project, the algorithm is being implemented in a simulator (Figure 17) because the car is still in the automation phase. This algorithm is intended to navigate the car in open areas, such as parking lots, while a different approach will be used to follow the lanes on roads.

## 8. Conclusions

A new path planning technique for mobile robots was successfully developed and tested, achieving up to a 56 percent decrease in planning time when compared with existing methods that are already quite efficient and have been optimized by various authors.

The pre-computation time can be very high, but this is done offline and can be greatly reduced using the techniques proposed here, such as the FFT method for cost maps in highly cluttered environments or the morphology dilation operation for clear binary maps.

In any case, the convolution of the whole map is done a single time, just after the occupancy grid generation. The resulting 3D map requires using more memory than previous approaches. Specifically, it requires *n* times more memory, being n=360/r, where *r* is the angular resolution used. For the warehouse map used in the Autoport project, this led to a 1.13 MB map, when a regular 2D occupancy grid map occupies 36.27 kB. For the tests performed on the willow SBPL map, the results were 65.69 MB against 4.10 MB. While this is a considerable increase from a percentual point of view, it is almost negligible considering today’s memory standards, and map sizes have no impact on the search time.

Replanning graph search algorithms can also be used with this technique, by calculating the convolution of newly found obstacles in real time. As these convolutions must be made for each layer of the 3D map, the impact of replanning will be higher than with other techniques that we have mentioned in this paper. In scenarios that require heavy replanning, this can offset the gains made by the planning time reduction, but using the convolution algorithms proposed here will reduce the computation time. In addition, in most situations, new obstacle discoveries are sporadic events.

## Figures and Tables

**Table 1:** Cubicle results.

Test	No. of Expanded Cells	Planning Time (s)	Avg. μs Per Cell	Solution Cost
Reference method	3,113,307	12.108	3.889	36,110
Proposed method	2,954,517	10.535	3.566	37,563

**Table 2:** Willow results.

Test	No. of Expanded Cells	Planning Time (s)	Avg. μs Per Cell	Solution Cost
Reference method	19,659,296	114.422	5.82	93,112
Proposed method	11,853,039	49.664	4.19	96,792

**Table 3:** Autoport project results.

Test	No. of Expanded Cells	Planning Time (s)	Avg. μs Per Cell	Solution Cost
Reference method	1,811,289	180.027	99.392	92.602
Proposed method	2,026,728	26.280	12.967	88.794

## Abbreviations

The following abbreviations are used in this manuscript:

RRT	Rapidly-exploring Random Tree
PRM	Probabilistic roadmap
MAXCOST	High value representing non traversable cells
FFT	Fast Fourier Transform
ARA*	Anytime Repairing A*
ADA*	Anytime Dynamic A*
SBPL	Search Based Planning Library
Ro-Ro	Roll-on and Roll-off
FEDER	Fondo Europeo Para el Desarrollo Regional (spanish for European Regional Development Funds)
MINECO	Ministerio de Economía y Competitividad (spanish ministry)
